# Accurate Quantification of Platelets and Leukocytes in Platelet-Rich Fibrin Using Direct Fibrinolytic Digestion: Effects of Centrifugation Protocols on Cellular Composition and Fibrin Architecture

**DOI:** 10.3390/biomedicines14051096

**Published:** 2026-05-13

**Authors:** Pattheera Apaiso, Wutigri Nimlamool, Teerada Daroontum, Supatra Sangin

**Affiliations:** 1 Department of Restorative Dentistry and Periodontology, Faculty of Dentistry, Chiang Mai University, Chiang Mai 50200, Thailand; goobgib.dt31@gmail.com; 2 Department of Pharmacology, Faculty of Medicine, Chiang Mai University, Chiang Mai 50200, Thailand; wutigri.nimlamool@cmu.ac.th; 3 Department of Pathology, Faculty of Medicine, Chiang Mai University, Chiang Mai 50200, Thailand; teerada.k@cmu.ac.th

**Keywords:** L-PRF, CGF, A-PRF, A-PRF+, direct quantification, fibrinolytic digestion, flow cytometry, fibrin architecture

## Abstract

**Background/Objectives**: Platelet-rich fibrin (PRF) is widely used in regenerative medicine and dentistry because of its ability to deliver growth factors and cellular components that support tissue healing. However, accurate quantification of platelets and leukocytes within PRF matrices remains challenging, as indirect subtraction-based methods may not reliably reflect the true cellular composition. This study aimed to apply a direct fibrinolytic digestion–flow cytometry approach for precise cellular quantification and to systematically evaluate the effects of different centrifugation protocols on PRF cellular composition and fibrin architecture. **Methods**: PRF clots were generated using high-speed protocols (leukocyte- and platelet-rich fibrin [L-PRF] and concentrated growth factors [CGF]) and low-speed protocols (advanced PRF [A-PRF] and A-PRF+). Cellular content was quantified using a fibrinolytic digestion-based approach with tissue plasminogen activator, followed by flow cytometry analysis. Histological evaluation was performed to assess fibrin architecture and cellular distribution. **Results**: L-PRF and CGF exhibited significantly greater platelet enrichment than A-PRF. T-lymphocyte counts were elevated across all PRF groups, with significantly higher levels observed in L-PRF and CGF than in A-PRF. Monocyte and neutrophil levels were reduced following PRF preparation; however, A-PRF retained significantly higher neutrophil levels than CGF, whereas B-lymphocyte levels showed a moderate increase without significant intergroup differences. Histologically, high-speed protocols produced pronounced cellular stratification and denser fibrin networks, whereas low-speed protocols demonstrated a more homogeneous leukocyte distribution within a porous fibrin matrix. **Conclusions**: Centrifugation protocols significantly influence PRF cellular composition and fibrin architecture. The direct fibrinolytic digestion–flow cytometry approach provides a reliable method for accurate cellular quantification and may facilitate the standardization and optimization of PRF preparation protocols for regenerative applications.

## 1. Introduction

Platelet-rich fibrin (PRF) is a biomaterial that has been widely utilized in various fields of medicine and dentistry for over three decades because of its capacity to deliver growth factors that stimulate wound healing and promote both soft- and hard-tissue regeneration [1]. Among these, leukocyte- and platelet-rich fibrin (L-PRF), introduced in 2001, represents a second-generation PRF produced through a single centrifugation step without the addition of anticoagulants [2,3,4]. During centrifugation, platelet activation occurs upon contact with the tube surface, initiating slow fibrin polymerization and leading to the formation of a three-dimensional fibrin scaffold. This fibrin scaffold acts as a reservoir for platelets, leukocytes, and growth factors, enabling their sustained release and thereby supporting cellular proliferation, migration, angiogenesis, extracellular matrix formation, and tissue repair [1,4]. Owing to its simple preparation and broad clinical applicability, PRF has become an important adjunct in regenerative dentistry, particularly in periodontal and bone regeneration procedures, either as a stand-alone biomaterial or in combination with grafting materials [5,6,7,8].

Continuous development of centrifugation techniques has led to several PRF derivatives designed to optimize cellular composition and biological activity. In 2014, advanced platelet-rich fibrin (A-PRF) was introduced based on the low-speed centrifugation concept (LSCC), which reduces relative centrifugal force (RCF) while extending centrifugation time (~200 g for 14 min) to enhance the distribution of platelets and leukocytes within the fibrin matrix [9,10]. A subsequent modification, A-PRF+, further shortened the centrifugation time while maintaining low centrifugal force (~200 g for 8 min), resulting in improved cellular preservation and increased fibrin matrix porosity [11,12,13]. Another derivative, concentrated growth factors (CGF), was developed using a centrifugation protocol with automatically varying rotational speeds (~692–855 g for 13 min), producing a denser and mechanically stronger fibrin matrix that may enhance the entrapment of cells and growth factors [14,15]. Because centrifugation parameters influence fibrin architecture and cellular distribution, different preparation protocols generate platelet concentrates with distinct cellular compositions and release profiles of growth factors, cytokines, and chemokines. A previous study has demonstrated that the amount of growth factor released is closely associated with the number of platelets and leukocytes retained within the fibrin scaffold [16].

Platelets and leukocytes entrapped within the PRF matrix play complementary roles in tissue repair. Activated platelets regulate early hemostasis and release key growth factors—including platelet-derived growth factor (PDGF), transforming growth factor-β (TGF-β), fibroblast growth factor (FGF), and vascular endothelial growth factor (VEGF)—that coordinate cellular proliferation, chemotaxis, extracellular matrix synthesis, angiogenesis, and tissue remodeling [4,17,18]. Leukocytes contribute additional cytokines and growth factors and interact with platelet-derived mediators through complex cellular crosstalk during wound healing [19]. Although the inclusion of leukocytes in platelet biomaterials has been debated because of their association with inflammatory responses, increasing evidence indicates that they also play important roles in immune regulation and tissue regeneration.

Despite the growing clinical use of PRF derivatives, accurate determination of platelet and leukocyte content within solid fibrin matrices remains a methodological challenge. Traditionally, cellular content has been estimated using indirect subtraction methods based on analysis of the liquid fractions remaining after clot formation, including serum, clot exudate, and red blood cells [20,21,22]. However, because these methods do not directly quantify cells incorporated into the fibrin matrix, they may underestimate the true cellular composition of solid PRF. Previous investigations have reported limited accuracy of subtraction techniques and their inability to reliably detect protocol-dependent differences in cellular retention [23]. To overcome these limitations, direct analytical approaches have been proposed to quantify cellular components within PRF matrices. Methods based on fibrinolytic digestion of the clot using tissue plasminogen activator (t-PA), followed by direct cell quantification using flow cytometry, enable the release and accurate quantification of platelets and leukocytes entrapped within the fibrin scaffold [24,25]. The primary advantage of the fibrinolytic digestion–flow cytometry approach lies in its ability to directly quantify platelets and leukocytes entrapped within the PRF matrix. This method overcomes the inherent limitations of indirect subtraction-based techniques, which may underestimate the actual cellular content. Furthermore, experimental studies employing such methods have demonstrated that direct analysis provides a more reliable assessment of the true cellular composition of platelet concentrates and enables meaningful comparisons between different centrifugation protocols.

However, comparative investigations of the cellular composition of different PRF derivatives using direct analytical methods remain limited. Therefore, the present study aimed to apply a direct fibrinolytic digestion–flow cytometry approach for precise cellular quantification and to systematically evaluate the effects of different centrifugation protocols on PRF cellular composition and fibrin architecture.

## 2. Materials and Methods

### 2.1. Subjects and Sample Collection

This study was approved on 23 September 2020 by the Human Experimentation Committee of the Office of Research Ethics, Faculty of Dentistry, Chiang Mai University (Approval No. 70/2020), and was conducted in accordance with the 1964 Helsinki Declaration and its later amendments.

Participants were voluntarily recruited from the dental staff of the Faculty of Dentistry, Chiang Mai University. Prior to sample collection, all participants were informed of the purpose of the study, and written informed consent was obtained. The inclusion criterion was healthy non-smoking volunteers aged 20–40 years. The exclusion criteria included: (i) systemic diseases such as diabetes mellitus or a recent history (within 6 months) of myocardial infarction (MI), cerebrovascular accident (CVA), immunocompromised conditions, chemotherapy, or radiotherapy; (ii) hematological disorders or bleeding abnormalities (e.g., platelet dysfunction or thrombocytopenia); (iii) use of anticoagulant or antiplatelet medication within the past 6 months; (iv) pregnancy or lactation; and (v) alcohol consumption. A total of six healthy volunteers (three males and three females) who met the inclusion criteria were enrolled, and the same individuals participated in both parts of the study.

### 2.2. Preparation of Platelet Concentrates

Blood samples were obtained from the median cubital vein under aseptic conditions using a 24-gauge butterfly needle. Each participant underwent two separate blood collections at three-month intervals, with 50 mL of blood drawn per session and transferred into 10 mL collection tubes designated for each of the four standard centrifugation protocols. Each individual donor was considered the primary experimental unit, while multiple PRF preparations derived from the same donor were treated as repeated measures to avoid pseudoreplication. The collected samples (five tubes of 10 mL each) were immediately centrifuged according to their assigned protocols to generate the L-PRF, CGF, A-PRF and A-PRF+. To ensure proper balance during centrifugation, 10 mL water-filled tubes were placed symmetrically opposite each sample tube in the rotor.

L-PRF clots were prepared from 10 mL of blood collected in a silica-coated plastic tubes (BD Vacutainer^®^, Becton Dickinson, Franklin Lakes, NJ, USA) and immediately centrifuged using the IntraSpin^TM^ PRF centrifuge (Intra-Lock, Boca Raton, FL, USA) at 2700 rpm (~708× *g* RCF max (~408× *g* RCF clot) for 12 min.

CGF clots were prepared in a similar manner, using 10 mL of blood collected in a silica-coated plastic tubes and immediately centrifuged with the Medifuge^®^ centrifugation device (Silfradent S. r. l., Santa Sofia, Italy) for 12 min, using the following program: 30 s acceleration, 2 min at 2700 rpm, 4 min at 2400 rpm, 4 min at 2700 rpm, 3 min at 3000 rpm, followed by 36 s deceleration and stop.

A-PRF and A-PRF+ clots were prepared from 10 mL of blood collected in vacuum plain glass tubes (A-PRF+^®^, Jiangxi Fenglin Medical Technology Co. Ltd., Fengcheng, China) using the DUO Quattro^TM^ centrifuge (Process for PRF, Nice, France). A-PRF samples were immediately centrifuged at 1300 rpm (~408× *g* RCF max (~208× *g* RCF clot)) for 14 min, whereas A-PRF+ samples were centrifuged at the same rpm for 8 min. The centrifugation protocols and devices are presented in Table 1.

**Figure 1:**
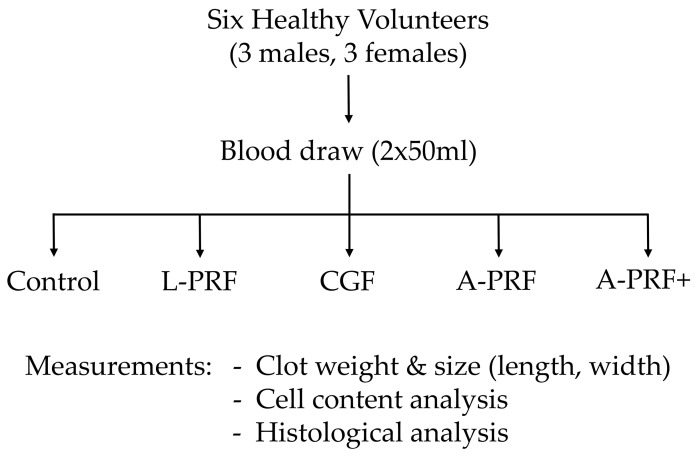
Flow diagram illustrating the overall study design and experiment workflow.

Following centrifugation, PRF clots were carefully retrieved using sterile tweezers and a smooth spatula to separate the red blood cell layer from the fibrin matrix. Each clot was placed on a sterile microscope slide, weighed, and its length and width were measured using a Vernier caliper. Clot weight and size were compared across all protocols, and representative samples were subsequently processed for cellular content and histological analyses. The experimental workflow is illustrated in Figure 1.

### 2.3. Digestion with Tissue Plasminogen Activator

The four PRF preparations (L-PRF, CGF, A-PRF, and A-PRF+) were digested using recombinant tissue plasminogen activator (t-PA) (Actilyse^®^; Boehringer Ingelheim Pharma GmbH & Co. KG, Ingelheim am Rhein, Germany). Lyophilized alteplase was reconstituted in the supplied water for injection to obtain a stock concentration of 6 × 10^5^ IU/mL immediately before use. Each sample was then mixed with 1 mL of the t-PA solution to achieve a final concentration of 2 × 10^5^ IU/mL and incubated at 37 °C for 24 h to ensure complete fibrin degradation. Following digestion, 50 µL of each lysate was stained with PE-conjugated anti-human CD41 antibodies (Beckman Coulter, Brea, CA, USA; BioLegend, San Diego, CA, USA) and incubated in the dark at room temperature for 15–20 min. The samples were subsequently lysed with 250 µL of OptiLyse^®^ C (Immunotech, Marseille, France) for an additional 15–20 min, diluted with 500 µL of phosphate-buffered saline (PBS), and analyzed by flow cytometry. For peripheral blood cell profiling, 50 µL of whole blood was stained with monoclonal antibodies against CD3, CD14, CD15, CD20, and CD45 (Beckman Coulter; BioLegend). After thorough vortexing, the samples were incubated in the dark for 15–20 min, lysed with OptiLyse^®^ C, diluted with PBS, and analyzed by the same flow-cytometry protocol.

### 2.4. Flow Cytometry for Platelet Quantification

For platelet analysis, 50 µL of whole blood was transferred into a 5 mL tube. In addition, 50 µL of supernatant and residual blood collected from each tube after clot removal were aliquoted into separate 5 mL tubes. Each sample was incubated with 5 µL of phycoerythrin (PE)-labeled anti-human CD41 monoclonal antibody (Beckman Coulter, Indianapolis, IN, USA), gently vortexed to ensure adequate mixing, and incubated in the dark at room temperature for 15–20 min. Subsequently, 250 µL of OptiLyse^®^ C (Immunotech, Marseille, France) was added to lyse red blood cells, followed by gentle mixing and further incubation in the dark at room temperature for 15–20 min. After lysis, 500 µL of phosphate-buffered saline (PBS) was added to each sample prior to analysis. All samples were analyzed using a CytoFLEX flow cytometer (Beckman Coulter, Indianapolis, IN, USA). A sequential gating strategy was applied, beginning with the exclusion of debris based on forward- and side-scatter characteristics (FSC-A vs. SSC-A), followed by identification of platelet populations based on CD41^+^ expression in combination with scatter properties. Fluorescence compensation was performed using single-stained controls for each fluorochrome, and compensation matrices were established prior to data acquisition. Appropriate unstained and/or isotype controls were included to define background fluorescence and marker positivity. To minimize platelet aggregation and doublet formation, samples were prepared under controlled conditions with gentle handling and appropriate dilution. Obvious cellular aggregates were excluded based on forward- and side-scatter (FSC/SSC) characteristics during gating. Following t-PA-mediated fibrin digestion, samples were gently resuspended to maintain cellular integrity prior to flow cytometric analysis. Complete fibrinolysis was confirmed by visual assessment, including the absence of visible clot remnants and the formation of a homogeneous cell suspension. In addition, consistent and well-defined flow cytometric profiles across samples supported efficient cellular release without substantial selective loss or damage.

### 2.5. Flow Cytometry for White Blood Cell Quantification

For leukocyte analysis, each sample was stained with a panel of monoclonal antibodies comprising 5 µL allophycocyanin (APC)-labeled anti-human CD3, 5 µL phycoerythrin (PE)-labeled anti-human CD14, 2.5 µL Pacific Blue-labeled anti-human CD15, 5 µL PC5-labeled anti-human CD20, and 5 µL ECD-labeled anti-human CD45 (all from Beckman Coulter, Indianapolis, IN, USA). Samples were gently vortexed to ensure uniform staining and incubated in the dark at room temperature for 15–20 min. Following incubation, 500 µL of phosphate-buffered saline (PBS) was added to each tube prior to analysis. Samples were analyzed using a CytoFLEX flow cytometer (Beckman Coulter, Indianapolis, IN, USA) for enumeration of leukocyte populations. A sequential gating strategy was applied, beginning with the exclusion of debris based on FSC-A vs. SSC-A characteristics, followed by identification of leukocyte populations using CD45^+^ gating. Leukocyte subpopulations were subsequently characterized using lineage-specific markers, including CD3 (T lymphocytes), CD14 (monocytes), CD15 (neutrophils), and CD20 (B lymphocytes). Fluorescence compensation was performed using single-stained controls for each fluorochrome, and compensation matrices were established prior to data acquisition. Appropriate unstained and/or isotype controls were included to define background fluorescence and marker positivity. To minimize doublet formation, samples were prepared under controlled conditions with gentle handling and appropriate dilution. Obvious cellular aggregates were excluded based on forward- and side-scatter (FSC/SSC) characteristics during gating. Following t-PA-mediated fibrin digestion, samples were gently resuspended to preserve cellular integrity prior to flow cytometric analysis. Complete fibrinolysis was confirmed by the absence of visible clot remnants and the formation of a homogeneous cell suspension, with consistent flow cytometric profiles supporting effective cellular recovery.

### 2.6. Histological Analysis

Fifty microliters of blood from each tube were aliquoted into separate tubes. The four types of PRF clots were fixed in 10% neutral-buffered formalin for 24 h. The specimens were then dehydrated through a graded ethanol series (60%, 70%, 80%, 90%, and 100%), cleared in xylene, and embedded in paraffin using an automatic tissue processor (Leica TP1020, Wetzlar, Germany). Paraffin blocks were sectioned at a thickness of 4–6 µm using a microtome. The tissue sections were deparaffinized by heating at 55 °C, followed by immersion in xylene, and subsequently rehydrated through a descending alcohol series. The rehydrated sections were stained with hematoxylin for 15 min, rinsed in water, and counterstained with eosin for 2 min. After staining, the slides were dehydrated again through graded alcohols, cleared in xylene, and mounted with coverslips. Histological evaluation was performed using a light microscope to assess the microscopic distribution of cellular components within the PRF clots. Histological images were captured with an Aperio AT Turbo Scanner (Copyright^©^ 2013 Leica Biosystems Imaging, Inc., Vista, CA, USA) at 20× magnification. Thereafter, the images were visualized using Aperio ImageScope software (version 12).

### 2.7. Statistical Analysis

Statistical analyses were performed using SPSS version 17 (SPSS Inc., Chicago, IL, USA). Descriptive statistics, including mean and standard deviation, were calculated for clot weight, clot size, and platelet and white blood cell counts across the four PRF preparation protocols. Differences among PRF protocols were analyzed using repeated-measures analysis of variance (ANOVA). Prior to analysis, data distribution was assessed for normality using the Shapiro–Wilk test, and the assumption of sphericity was evaluated using Mauchly’s test. When the assumption of sphericity was violated, the Greenhouse–Geisser correction was applied. Post hoc pairwise comparisons were performed using Bonferroni-adjusted tests to account for multiple comparisons and control the type I error rate. A two-tailed *p*-value < 0.05 was considered statistically significant.

## 3. Result

### 3.1. Macroscopic Characteristics of PRF Clots

**Figure 2:**
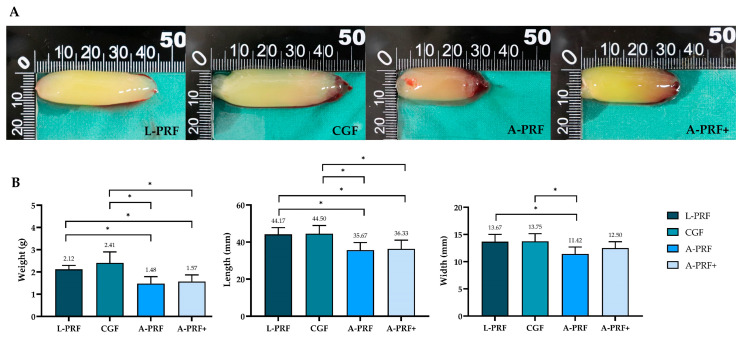
Macroscopic analysis of PRF clots prepared using high- and low-speed protocols. Representative images show (**A**) clot morphology, (**B**) mean clot weight, clot length, and clot width. Data are presented as mean ± standard deviation (SD), and statistically significant differences are indicated by * (*p* < 0.05).

The macroscopic characteristics of fibrin clots generated by the high- and low-speed centrifugation protocols were evaluated by measuring clot weight, length, and width. Representative macroscopic images of the four PRF clots (L-PRF, CGF, A-PRF, and A-PRF+) are shown in Figure 2A. The clots exhibited clear differences in size among the centrifugation protocols. Quantitative analysis demonstrated significant differences in clot weight, length, and width among the groups (Figure 2B). Overall, the high-speed protocols produced significantly heavier and larger clots than the low-speed protocols, whereas no significant difference in clot width was observed between the high-speed protocols and A-PRF+. Regarding clot size, no significant differences were observed between the two high-speed groups (L-PRF, CGF) or between the two low-speed groups (A-PRF, A-PRF+).

### 3.2. Platelet Quantification

**Figure 3:**
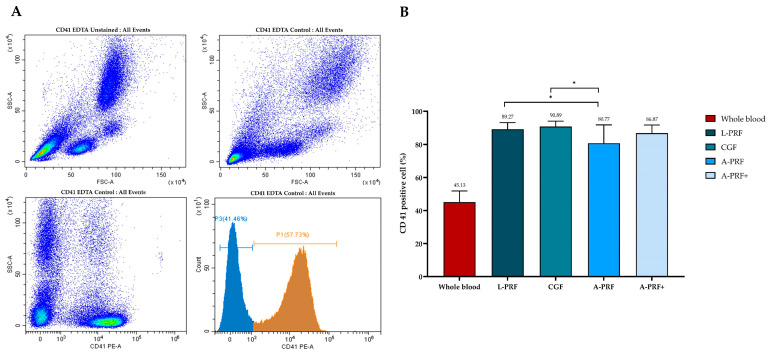
Platelet quantification in whole blood and PRF clots. Representative images show (**A**) flow cytometry gating strategy used to identify CD41^+^ platelet populations and (**B**) percentage of CD41^+^ platelets in whole blood and PRF clots prepared using L-PRF, CGF, A-PRF, and A-PRF+ protocols. L-PRF and CGF demonstrated significantly higher platelet percentages than A-PRF (*p* < 0.05). Data are presented as mean ± standard deviation (SD), and statistically significant differences are indicated by * (*p* < 0.05).

Platelet content in PRF clots was quantified using flow cytometry. A representative gating strategy for the identification of viable single cells and CD41^+^ platelet populations is presented in Figure 3A. The baseline platelet percentage in whole blood was 45.13 ± 6.74%. Following centrifugation, all PRF preparations demonstrated significantly higher platelet percentages than whole blood (*p* < 0.05). Specifically, CD41^+^ platelet percentages were 89.27 ± 4.00% in L-PRF, 90.89 ± 3.28% in CGF, 80.77 ± 11.12% in A-PRF, and 86.87 ± 4.93% in A-PRF+.

Comparative analysis revealed that L-PRF and CGF exhibited significantly higher platelet enrichment than A-PRF (Figure 3B), whereas no significant differences were observed among L-PRF, CGF, and A-PRF+. Overall, these findings indicate that all centrifugation protocols effectively concentrate platelets relative to whole blood, with high-speed protocols yielding the greatest platelet enrichment.

### 3.3. White Blood Cells Quantification

**Figure 4:**
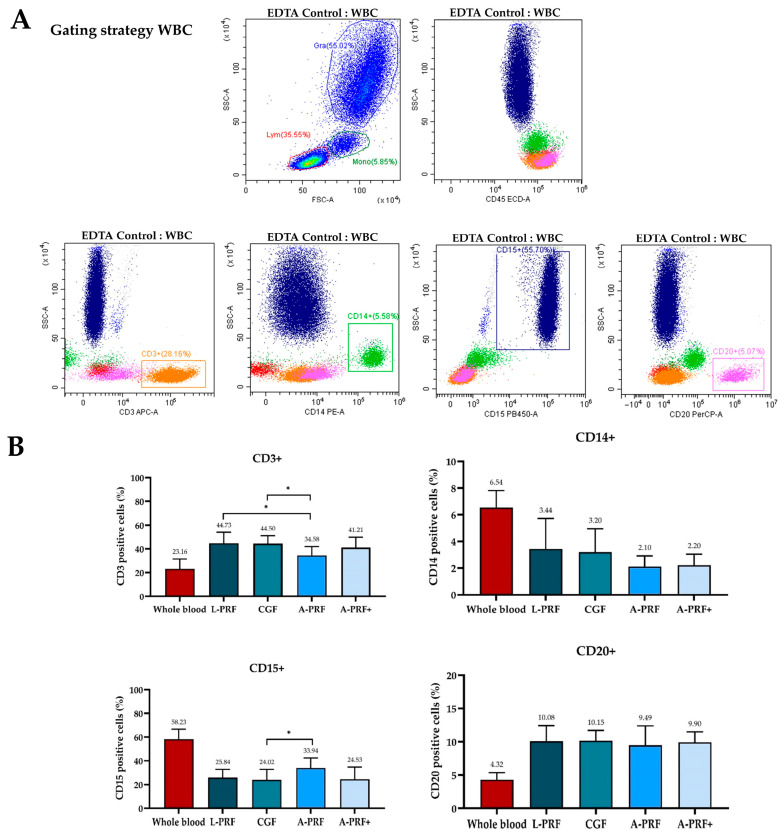
White blood cell quantification in whole blood and PRF clots. Representative images show (**A**) flow cytometry gating strategy used to identify leukocyte subpopulations and (**B**) percentages of CD3^+^ T lymphocytes, CD15^+^ neutrophils, CD20^+^ B lymphocytes, and CD14^+^ monocytes in whole blood and PRF prepared using L-PRF, CGF, A-PRF, and A-PRF+ protocols. Data are presented as mean ± standard deviation (SD), and statistically significant differences are indicated by * (*p* < 0.05).

Flow cytometric analysis of white blood cell subpopulations is presented in Figure 4 and demonstrates distinct differences in leukocyte composition between whole blood and PRF preparations. T lymphocytes (CD3^+^) were increased in all PRF groups compared with whole blood, with significantly higher proportions observed in L-PRF and CGF than in A-PRF. In contrast, monocytes (CD14^+^) were consistently reduced following PRF preparation, with no significant differences among centrifugation protocols. Neutrophils (CD15^+^), which predominated in whole blood, were markedly decreased in all PRF matrices; however, A-PRF demonstrated significantly higher neutrophil levels than CGF. B lymphocytes (CD20^+^) were moderately increased in PRF clots relative to whole blood, with no significant differences observed among the PRF protocols. Overall, these findings indicate that PRF preparation modulates leukocyte composition in a protocol-dependent manner, with high-speed protocols favoring lymphocyte enrichment and low-speed protocols preserving a relatively higher proportion of granulocytes.

### 3.4. Histological Analysis

Histological evaluation using hematoxylin and eosin staining demonstrated clear protocol-dependent differences in cellular distribution and fibrin architecture among PRF preparations. In L-PRF and CGF, leukocytes—predominantly polymorphonuclear cells—were densely concentrated within the lower (buffy coat) region, with platelets localized at the fibrin–erythrocyte interface. This region was characterized by a compact and highly organized fibrin network that progressively transitioned into a looser structure toward the upper portion of the clot. In contrast, A-PRF and A-PRF+ exhibited a more homogeneous distribution of both polymorphonuclear and mononuclear leukocytes, extending beyond the lower region into the middle layer of the fibrin matrix. This distribution was associated with a fibrin architecture that, although structurally dense, appeared more porous and less compact compared with high-speed protocols. Across all PRF types, the upper region consisted predominantly of fibrin with scattered platelets and an absence of detectable leukocytes.

**Figure 5:**
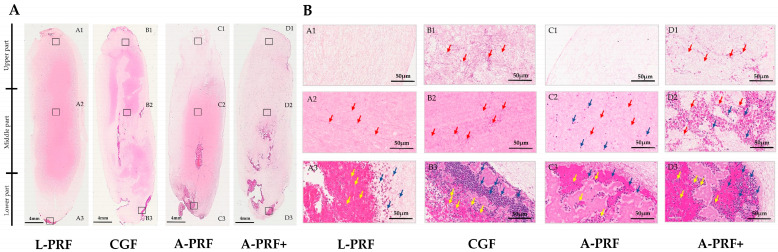
Histological features of PRF clots prepared using high- and low-speed centrifugation protocols. Representative hematoxylin and eosin-stained sections show (**A**) whole-clot longitudinal morphology and (**B**) regional cellular distribution and fibrin architecture in the upper, middle, and lower regions of PRF clots. Scale bar = 50 µm. Yellow arrows indicate polymorphonuclear white blood cells; blue arrows indicate mononuclear white blood cells and red arrows highlight the morphology of platelets.

Overall, high-speed protocols (L-PRF and CGF) demonstrated pronounced cellular stratification and a compact fibrin architecture, whereas low-speed protocols (A-PRF and A-PRF+) showed a more uniform cellular distribution within a comparatively porous fibrin network. A representative comparison of the upper, middle, and lower regions across all PRF types is presented in Figure 5.

## 4. Discussion

Over the past decade, PRF has emerged as promising regenerative biomaterial due to their fibrin scaffold and sustained release of growth factors. The biological quality of PRF is strongly influenced by centrifugation parameters, which regulate fibrin architecture, cellular distribution, and growth factor release dynamics. Previous studies have shown that variations in relative centrifugal force, centrifugation time, and centrifuge configuration significantly affect fibrin network organization, cellular retention, and cytokine release profiles [26]. In addition, differences in cellular composition have been directly associated with variations in growth factor secretion [16]. Collectively, these findings underscore the critical role of centrifugation parameters in determining both the structural and biological characteristics of PRF, highlighting the need for accurate and standardized cellular characterization across preparation protocols. However, the influence of centrifugation on cellular composition remains controversial, largely because of methodological variability, particularly in the quantification of platelets and white blood cells within solid fibrin matrices. This study aimed to directly quantify and histomorphologically characterize platelet and leukocyte content in PRF clots prepared using high- and low-speed centrifugation protocols. To our knowledge, few studies have systematically compared cellular composition across multiple platelet concentrate formulations using direct quantification methods. Therefore, a fibrinolytic digestion-based approach combined with flow cytometry and histomorphological analysis was employed to accurately assess platelets and leukocytes entrapped within the insoluble fibrin matrix.

The findings demonstrate that centrifugation protocols significantly influence PRF quality, affecting clot dimensions, fibrin architecture, and cellular composition. A-PRF exhibited the smallest clot size, with statistically significant differences compared with L-PRF and CGF. These findings are consistent with previous studies reporting that A-PRF and A-PRF+ generally present smaller clot sizes than L-PRF and CGF [27,28,29]. However, our results contrast with studies reporting no significant differences in size and weight between A-PRF and other fibrin clots [17,30,31]. Mechanistically, higher-speed centrifugation accelerates fibrin polymerization and promotes tighter fibrin fiber packing, resulting in compact and densely organized clots. In contrast, LSCC protocols, allow slower fibrin polymerization and the formation of a more porous and loosely organized fibrin network. These structural differences are biologically relevant, as fibrin density and porosity influence cellular retention, growth factor diffusion, and cell–matrix interactions within PRF matrices [11,12,32]. Previous studies have demonstrated that donor-specific factors, including gender, age, hematocrit, fibrinogen levels platelet count, and platelet function, may significantly influence PRF clot size and structural characteristics [33,34]. In particular, lower hematocrit levels—more frequently observed in females—have been associated with the formation of larger L-PRF membranes, likely because of reduced red blood cell volume and altered clot stratification during centrifugation. Furthermore, differences in fibrinogen levels may influence fibrin network density and resistance to fibrinolysis, thereby contributing to variability in clot structure and biological activity [34]. Collectively, these observations highlight the importance of donor-related biological variability in determining PRF characteristics. Although all donors in the present study exhibited normal hematological parameters, inter-individual variability cannot be entirely excluded and may have contributed to the observed differences in clot properties.

In the present study, platelet quantification using CD41 demonstrated that A-PRF exhibited the lowest platelet counts, followed by A-PRF+, whereas L-PRF and CGF showed comparatively higher platelet enrichment within the fibrin clots. These findings are consistent with previous reports indicating that increased relative centrifugal force enhances platelet sedimentation and incorporation into more densely organized fibrin scaffolds [23,28,35]. However, our results contrast with the LSCC, which proposes that reduced RCF promotes greater cellular retention within the PRF matrix. Discrepancies in the literature may be partly explained by methodological limitations. Previous studies have shown that the commonly used subtraction method may overestimate platelet counts due to platelet contamination within the red thrombus fraction and adhesion to fibrin matrices and tube surfaces [23]. Furthermore, some investigations have reported no significant differences in platelet and leukocyte counts between high- and low-speed protocols [17,23], suggesting that analytical variability and experimental conditions influence the interpretation of cellular composition in PRF matrices.

The impact of centrifugation parameters on PRF cellular content remains controversial. Studies supporting the LSCC [9,12] have reported increased cellular presence or growth factor release under reduced RCF; however, these conclusions are largely based on indirect subtraction-based quantification or qualitative histological assessments, which may not accurately reflect the total number of platelets incorporated within the PRF matrix. In contrast, studies examining centrifugation physics and fibrin architecture have indicated that higher RCF promotes cellular sedimentation and compaction [27,28]. These findings are consistent with the present study, which demonstrated significantly higher absolute platelet counts in high-speed protocols than in low-speed protocols. This apparent discrepancy may be attributed to differences in analytical methodology and outcome interpretation. Mechanistically, higher RCF may enhance cellular sedimentation toward red blood cell (RBC)-associated regions, thereby increasing the absolute incorporation of platelets into the fibrin clot, particularly at the red thrombus interface. This suggests that the measured platelet yield is strongly influenced by clot harvesting and recovery methods. Despite efforts to exclude the red thrombus during clot collection, even minimal inclusion of this platelet-dense proximal layer may have contributed to the elevated platelet counts observed in high-speed protocols. Furthermore, higher RCF produces a denser fibrin architecture, which may enhance mechanical entrapment of platelets within the proximal clot region. Conversely, lower-RCF are likely to produce a more porous fibrin network that favors cellular redistribution rather than maximal platelet compaction. This structural difference may promote biological activity, such as growth factor release or cell migration, without necessarily increasing the total platelet yield. Collectively, these findings indicate that absolute platelet yield and spatial cellular distribution represent distinct biological parameters that should not be interpreted interchangeably. Accordingly, it is important to distinguish between total cellular content and spatial organization within PRF matrices, as the LSCC may primarily influence cell localization and functional dynamics rather than overall platelet quantity.

Analysis of leukocyte subpopulations demonstrated that centrifugation protocols influence the cellular composition of PRF matrices. T lymphocytes (CD3^+^) represented a major leukocyte population within fibrin clots and were present in significantly higher proportions in L-PRF and CGF than in A-PRF, suggesting that higher centrifugal forces favor lymphocyte enrichment through enhanced cellular entrapment within denser fibrin networks [33]. In contrast, low-speed protocols such as A-PRF preserved relatively higher proportions of neutrophils (CD15^+^), likely because of reduced sedimentation forces that facilitate greater retention of granulocytes within the fibrin matrix. This finding supports the concept that low-speed centrifugation favors preservation of innate immune cells [9,26]. B lymphocytes (CD20^+^) showed moderately increased proportions in PRF compared with whole blood but did not differ significantly among centrifugation protocols. Although present in smaller proportions, B cells may contribute to immune regulation and cytokine signaling within PRF matrices. Monocytes (CD14^+^) were consistently reduced across all PRF preparations relative to whole blood, with no significant differences observed between protocols, consistent with previous investigations reporting no significant differences in leukocyte subset frequencies among protocols [16,17,23]. Despite their lower abundance, monocytes remain biologically important because they can differentiate into macrophages that regulate angiogenesis, immune modulation, and tissue remodeling during wound healing. The consistent reduction observed across PRF types suggests selective redistribution during centrifugation and fibrin polymerization. Due to their intermediate density and size, monocytes may preferentially localize near the buffy coat interface or partially migrate toward the RBC fraction, resulting in relative depletion within the final clot matrix. Collectively, these findings confirm that PRF matrices are not merely platelet concentrates but biologically active constructs containing distinct leukocyte populations that vary according to centrifugation protocol. Although leukocytes are capable of secreting a wide range of cytokines and growth factors, their biological activity within PRF is strongly influenced by the surrounding fibrin matrix and interactions with platelets. The fibrin network provides a three-dimensional scaffold that supports cell retention, spatial organization, and the sustained release of bioactive molecules over time. Moreover, the structural properties of the fibrin matrix facilitate cell–cell interactions and regulate the temporal release of cytokines, thereby enhancing biological activity in a coordinated manner. Consequently, the regenerative potential of PRF arises from the dynamic interplay between its cellular and structural components.

Histomorphological analysis corroborated the quantitative findings. Light microscopic analysis (H&E staining) revealed that platelets and leukocytes were concentrated in the lower region adjacent to the erythrocyte layer across all PRF types, whereas the middle region contained fewer dispersed cells within densely aligned fibrin fibers, particularly in A-PRF and A-PRF+. The upper region was largely composed of loosely organized fibrin with sparse platelets and minimal leukocyte presence. These observations are consistent with previous reports indicating that higher centrifugal forces promote cellular stratification at the fibrin–erythrocyte interface, whereas reduced RCF facilitates a more homogeneous cellular distribution within the fibrin scaffold and the formation of a more porous network [9,12]. From a biological perspective, fibrin architecture is a critical determinant of PRF functionality, as matrix density and organization influence growth factor retention and release kinetics, cellular migration, and angiogenic potential. In the present study, high-speed protocols produced a dense and compact fibrin network with pronounced cellular stratification, whereas low-speed protocols resulted in a more porous architecture with broader leukocyte distribution throughout the matrix. Clinically, these findings have important implications for regenerative therapy, as understanding how centrifugation protocols affect cellular composition is essential for standardization and reproducibility.

The key advantage of the direct fibrinolytic digestion–flow cytometry approach lies in its ability to provide a robust and reliable method for quantifying cellular composition within PRF matrices, thereby overcoming the limitations of conventional subtraction-based techniques. By enzymatically degrading the fibrin network with t-PA, this method enables accurate enumeration of platelets and leukocytes entrapped within the fibrin network across different PRF centrifugation protocols. Previous studies have demonstrated that platelet quantification based on whole blood reference values and t-PA digestion provides comparable accuracy, with sensitivity and specificity similar to those of gold-standard methods [25]. In addition to improved analytical accuracy and reproducibility, this approach offers several practical advantages, including high precision, a relatively simple procedure, minimal technical skill requirements, and broad applicability across different centrifugation protocols [24,25]. However, it is associated with longer processing times, increased technical demands, and higher reagent costs compared with indirect estimation methods.

Despite the strengths of the present study, several limitations should be acknowledged. The sample size was relatively small, which may limit the generalizability of the findings. However, the study was designed to provide controlled mechanistic insights under standardized conditions rather than population-level inference. In addition, inter-individual donor variability, including differences in demographic characteristics and hematological parameters, may influence PRF composition and cannot be entirely excluded despite careful screening and standardized experimental conditions. This study primarily focused on ex vivo quantification of platelet and leukocyte content and histomorphological characterization of PRF clots, without direct assessment of growth factor release kinetics or functional biological activity.

Certain methodological aspects of the flow cytometry analysis also represent limitations. A dedicated doublet discrimination step (e.g., FSC-A vs. FSC-H gating) was not formally implemented, and a specific viability dye was not included. Although multiple orthogonal parameters—such as preserved FSC/SSC profiles and CD45^+^ gating—were used to support the analysis of intact cells, the absence of direct viability assessment may influence the accuracy of cellular characterization. In addition, although fibrinolytic digestion with t-PA enables accurate cellular quantification, enzymatic processing may not fully replicate in vivo fibrin degradation dynamics.

Moreover, the histological findings presented in this study are primarily descriptive in nature and should be interpreted with caution, as no quantitative histomorphometric analysis was performed to objectively assess structural differences. Furthermore, histological evaluation based on hematoxylin and eosin staining provides limited specificity for detailed characterization of fibrin architecture and cellular phenotypes compared with advanced techniques such as immunohistochemistry or scanning electron microscopy. Finally, variations in centrifuge systems, rotor angulation, blood collection tube materials, and device configurations—including differences in the calculation of relative centrifugal force (e.g., RCF-max vs. RCF-clot)—may act as confounding factors. Therefore, the observed differences should be interpreted as the combined effects of protocol-specific conditions rather than a single parameter, and such variability may complicate direct comparisons across studies.

While the present study provides comprehensive mechanistic insights into the cellular composition and fibrin architecture of PRF prepared using different centrifugation protocols, further research is required to translate these findings into clinically meaningful outcomes. Future investigations should focus on correlating in vitro cellular characteristics and spatial distribution with in vivo biological performance, including growth factor release kinetics and regenerative capacity. In addition, well-designed clinical trials are needed to evaluate the efficacy of different PRF preparation protocols in wound healing, soft tissue and bone regeneration, and patient-centered outcomes. Future studies incorporating larger sample sizes, standardized methodologies, inclusion of viability assessment, quantitative histomorphometric analysis, and evaluation of biological activity and growth factor release are warranted to validate and extend these findings. Ultimately, integrating quantitative cellular analysis with clinical evidence will be essential to establish standardized, evidence-based guidelines for PRF preparation and application in regenerative dentistry.

## 5. Conclusions

This study demonstrated that centrifugation protocols significantly influence the structural and cellular characteristics of platelet-rich fibrin matrices. High-speed protocols produced larger and heavier fibrin clots with greater platelet enrichment, whereas low-speed protocols resulted in comparatively smaller clots with broader leukocyte distribution throughout the fibrin matrix. Direct fibrinolytic digestion combined with flow cytometry provides a reliable approach for accurate cellular quantification and may support the standardization and optimization of PRF preparation protocols for regenerative applications.

## Figures and Tables

**Table 1:** Devices and centrifugation parameters used for each PRF preparation protocol.

	High-Speed Protocols		Low-Speed Protocols	
	L-PRF	CGF	A-PRF	A-PRF+
RCFmax	~708× *g*	~692× *g* ~547× *g* ~692× *g* ~885× *g*	~208× *g*	~208× *g*
RPM (time)	2700 (12 min)	2700 (2 min) 2400 (4 min) 2700 (4 min) 3000 (3 min)	1300 (14 min)	1300 (8 min)
Centrifuges	IntraSpin^TM^	Medifuge^®^	DUO Quattro^TM^	DUO Quattro^TM^
Rotor types (Angulation)	Angle (33°)	Angle (33°)	Angle (43.1°)	Angle (43.1°)
Vacuum tubes	BD Vacutainer^®^ (silica-coated)	BD Vacutainer^®^ (silica-coated)	A-PRF+^®^ (plain glass)	A-PRF+^®^ (plain glass)

## Data Availability

The original contributions presented in this study are included in the article. Further inquiries can be directed to the corresponding authors.

## Abbreviations

The following abbreviations are used in this manuscript:

APC	Autologous platelet concentrates
PRF	Platelet-rich fibrin
L-PRF	Leukocyte- and platelet-rich fibrin
CGF	Concentrated growth factors
A-PRF	Advanced platelet-rich fibrin
A-PRF+	Advanced platelet-rich fibrin plus
LSCC	Low-speed centrifugation concepts
t-PA	Tissue plasminogen activator
